# *Caenorhabditis elegans* exhibit a coupling between the defecation motor program and directed locomotion

**DOI:** 10.1038/srep17174

**Published:** 2015-11-24

**Authors:** Stanislav Nagy, Yung-Chi Huang, Mark J. Alkema, David Biron

**Affiliations:** 1 The Institute for Biophysical Dynamics, The University of Chicago, Chicago, IL; 2 Department of Neurobiology, University of Ma ssachusetts Medical School, Worcester, MA; 3 Department of Physics and the James Franck Institute, The University of Chicago, Chicago, IL

## Abstract

Distinct motor programs can be coupled to refine the repertoire of behavior dynamics. However, mechanisms underlying such coupling are poorly understood. The defecation motor program (DMP) of *C. elegans* is composed of a succession of body contraction and expulsion steps, performed repeatedly with a period of 50–60 sec. We show that recurring patterns of directed locomotion are executed in tandem with, co-reset, and co-terminate with the DMP cycle. Calcium waves in the intestine and proton signaling were shown to regulate the DMP. We found that genetic manipulations affecting these calcium dynamics regulated the corresponding patterns of directed locomotion. Moreover, we observed the initiation of a recurring locomotion pattern 10 seconds prior to the posterior body contraction, suggesting that the synchronized motor program may initiate prior to the DMP. This study links two multi-step motor programs executed by *C. elegans* in synchrony, utilizing non-neuronal tissue to drive directed locomotion.

Many animals couple locomotion-driven behaviors to defecation or to the presence of feces. A common example is fecal avoidance: reindeer, antelopes, livestock, primates, kangaroos and mice (under certain conditions) preferentially forage away from feces to reduce contact with parasites^1^,^2^,^3^,^4^,^5^,^6^,^7^. Among invertebrates, examples include species of ants that defecate in dedicated refuse sites^8^,^9^ and defecation flights of honeybees^10^,^11^. However, such a coupling of defecation to locomotion in nematodes has not been previously reported.

Spontaneous behaviors of the nematode *Caenorhabditis elegans*, such as locomotion or egg-laying, offer simple models for mechanisms that may be broadly conserved. Among these, the defecation motor program (DMP) provides an example of a well-studied rhythmic process. In well-fed animals, the DMP consists of a series of stereotypical steps that are executed repeatedly with a period of 50–60 seconds^12^,^13^. These steps, typically assayed by visual inspection, include the simultaneous contraction of dorsal and ventral posterior body-wall muscles (pBoc), the simultaneous contraction of dorsal and ventral anterior body-wall muscles (aBoc), and the contraction of enteric muscles resulting in the expulsion (Exp) of the gut contents concurrent with the aBoc^12^,^13^,^14^.

Molecular and cellular techniques have been used to identify the mechanisms that generate and maintain the periodicity of the DMP, and its dependence on food availability and temperature^12^,^15^,^16^. As a result, this rhythmic behavior provides a model for the genetics underlying behavioral rhythms, calcium oscillations, synaptic transmission, retrograde signaling, and regulation of behavior by non-neuronal tissue^12^,^13^,^17^,^18^,^19^,^20^,^21^.

Calcium waves in intestinal cells play a key role in the ultradian pacemaker of the DMP. A localized calcium peak originates in the posterior intestine and propagates towards the anterior^22^,^23^. The calcium induces pumping of protons from the intestinal cells to the pseudocoelomic space via the PBO-4/NHX-7 Na^+^/H^+^ exchanger. Acidification of the pseudocoelomic space activates PBO-5/6 proton-gated ion channels, which depolarize the overlaying body wall muscles to generate the pBoc^21^,^23^,^24^,^25^,^26^,^27^,^28^.

The intestinal calcium flux is required to reach the anterior for the execution of the aBoc and expulsion steps^23^,^24^,^26^: a pannexin intestinal gap junction subunit, INX-16, facilitates the progression calcium along the intestine^24^ and the GABAergic neuron AVL participates in controlling the aBoc once anterior calcium levels return to baseline^13^,^23^,^26^,^29^. Neuropeptides released from the intestine depolarize the GABAergic DVB motoneuron, which stimulates the contraction of the enteric muscle to produce expulsion^19^,^20^,^25^,^29^,^30^,^31^.

This study reports a recurring pattern of directed locomotion that is coupled to the DMP. Precise and continuous measurements of body-length enabled reliable automatic detection of body-wall muscle contractions and the alignment of the dynamics of posture and locomotion with the DMP cycle. The alignment of body-length and locomotion data revealed that *C. elegans* exhibited locomotion patterns that may assist the separation of feces from patches of food: increased propensities for backward locomotion just before expulsion and increased propensities for forward locomotion immediately after expulsion. We refer to this modulation of directed locomotion as the DMP associated motor program (DAMP).

We found that the intestine instructed the prominent peak of backward motion between the Bocs. In contrast, vesicle exocytosis from the interneuron types AVA and AVE, previously shown to promote backward locomotion^32^,^33^,^34^, were not strictly required for generating this peak. Proton signaling was found to play a role in establishing and shaping the dynamics of the DAMP. While the origin of the coupling between the DMP and the DAMP remains unclear, a simple hypothesis consistent with the data would invoke a single pacemaker initiating the two programs several seconds prior to the occurrence of the pBoc.

## Results

### Precise measurements of body length enable automated detection of the DMP body contractions

**Figure 1:**
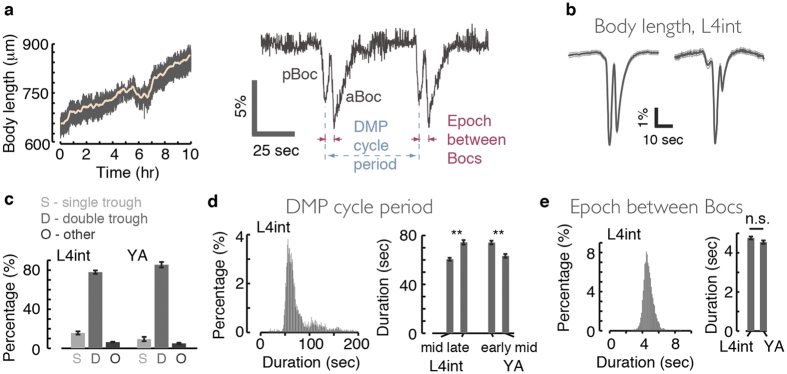
Precision measurements of body length enable automated measurements of the DMP. (**a**) Left: a continuous 10-hour record of the body-length of a single animal from the mid L4int stage, through L4leth, to the early YA stage. Light colored line depicts a running window average. Right: an enlarged view of 2 minutes of data demonstrates that the 4–6% fluctuations in the data do not originate from measurement noise, but rather correspond to changes in the body length as a result of the pBocs and aBocs. Measurements of the durations of the DMP cycle and epochs between Bocs are illustrated in blue and red, respectively. (**b**) The average body-length during the defecation cycle, aligned at the trough of the pBoc, of wild-type late L4 larvae. Left and right panels depict the mean body length (averaged over all animals assayed) of double- and single-trough events, respectively. (**c**) The rates at which one, two, or more body contractions were identified within a 10 sec window. (**d**) Left: the distribution of periods between successive DMP cycles. Right: the mean periods between defecation cycles during different developmental times. Before and after L4 lethargus the period of the cycle was elongated. (**e**) Left: the distribution of durations of epochs between body contractions within a DMP cycle. Right: the inter-Boc epoch did not change just prior to or after L4 lethargus. In all panels N = 28 animals and error bars or thin lines depict ± s.e.m. Double asterisks denote p < 0.01.

The body contractions and expulsion step associated with the defecation cycle of *C. elegans* are typically scored by visual inspection^12^,^13^,^14^,^15^,^31^. While this method facilitated much progress in understanding the genetics of the DMP, automated assays may be used to increase throughput and sensitivity. This would enable detection of subtle phenotypes and ensure unbiased scoring. Measurements of body-length in freely behaving animals provide a machine vision based approach for automated detection of pBocs and aBocs (Fig. 1a,b). Such measurements can be performed continuously for arbitrarily long periods, in highly controlled environments, with minimal disturbance to the animal.

To validate our machine vision approach, we first assayed wild-type animals. Each animal was assayed for 10 continuous hours, between the mid L4 intermolt (L4int) and mid young adult (YA) stages. The data presented in this manuscript was obtained from L4 larvae unless stated otherwise. However, in all cases where high quality data from young adults was available, we did not observe differences between the two stages. A contraction event was scored if one or more troughs in body-length were detected within a short period (see methods). The events were then divided between three categories: pairs of adjacent troughs typical of the DMP, single troughs, or multiple troughs.

Figure 1c depicts the frequencies of the detected events in each category. Double contractions were detected in 80% of the cases, while 15% of events were scored as single contractions. Either misidentification or a body contraction that was truly missing could potentially result in detecting a single Boc. Visual inspection revealed sub-threshold contractions in the majority of these cases (see also Fig. 1b, right panel). Previously studied aBoc or pBoc deficient mutants^12^,^13^,^20^,^31^,^35^,^36^ exhibited their respective reported phenotypes in our assay (Fig. S1). Consistent with the known aBoc defect of *unc-31* mutants, deficient in peptidergic signaling^36^, we also found that loss of function of the proprotein convertase EGL-3/KPC-2 or carboxypeptidase E EGL-21 abolished aBocs (Fig. S1).

The recorded timing of many contraction events revealed the distributions of the two timescales characteristic of the DMP: the period of the cycle and the duration of the epoch between the Bocs. In agreement with previous findings^12^, the cycle period was 60 sec during the mid L4 and mid YA stages. However, this period was extended shortly before and after L4 lethargus, a stage in which feeding and defecation are suspended (Fig. 1d). In contrast, the durations of the intra-cycle epochs remained 4.5 sec throughout our assays (Fig. 1e).

It has been shown that protein kinase A (PKA) activates the GABAergic neurons responsible for enteric muscle contraction in response to the peptidergic signal from the intestine^31^,^37^. While constitutively active PKA was observed to result in ectopic expulsions and to extend the duration of calcium transients in the DVB motoneuron^31^,^37^, its effect on the timing of the DMP steps was not reported. We observed a significant extension of the epoch between the Bocs, which mirrored the extension of calcium transients in DVB (Fig. S2). Collectively, these results validated the method, demonstrated its advantages, and revealed that the DMP period is modulated during development.

### The DMP is coupled to motor program which coordinates directed locomotion

*C. elegans* move directionally by propagating dorsoventral undulations along their body from the anterior to the posterior or vice versa. Non-directional body movements are collectively referred to as dwelling behavior. Thus, locomotion is implemented via the temporal modulation of body-posture^38^,^39^,^40^. Since the same muscle groups underlie DMP body contractions and locomotion, we asked whether the two motor programs might be coordinated. To address this, we performed an analysis of forward, backward, and non-directional (dwelling) locomotion as previously described^41^. Combining the automatic detection of body contractions with this analysis enabled the alignment of posture and locomotion dynamics with the peak of the pBoc (defined as t = 0 unless stated otherwise) and averaging over thousands of DMP cycles.

**Figure 2:**
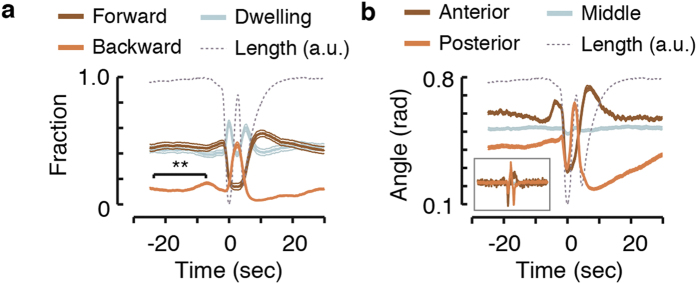
The DMP is coupled to a stereotypical locomotion motor program. (**a**) The likelihood of observing forward locomotion, backward locomotion, and dwelling during a DMP cycle. For each DMP cycle, locomotion data were aligned at the trough of the pBoc (t = 0) before averaging. The observed coordinated locomotion cycle is referred to as the DAMP. (**b**) The mean magnitude of angles (corresponding to local curvature – see methods) measured at three regions along the midline of the animal: the most anterior, the mid-body, and the most posterior. Data were aligned at the trough of the pBoc (t = 0) before averaging. Inset: the mean rates of change of the anterior and posterior angles. Dotted grey lines in panels (**a**,**b**) represent body-length in arbitrary units as a guide to the eye.

Interestingly, locomotion during the DMP cycle was coordinated in a reproducible manner (Fig. 2a). Backward locomotion mildly peaked 8 seconds before the pBoc, prominently peaked between the pBoc and the aBoc, and was suppressed just prior to the pBoc and during and after the aBoc. Complementarily, the likelihood of forward locomotion was suppressed between the Bocs and transiently elevated following expulsion. The small but significant elevation in backward motion observed prior to the pBoc also preceded the reported timing of the onset of calcium transients in the intestine^23^,^24^,^26^. As indicated by the amplitude of the peak in Fig. 2a, backward locomotion between the Bocs was detected in half of the DMP cycles under our experimental conditions. Animals thus preferentially briefly retreated before the expulsion step and moved forward afterwards (see supplementary movie M1). We refer to this coordinated quasi-cycle as the DMP associated motor program (DAMP).

The body posture of *C. elegans* also exhibited stereotypical modulation during the DMP (Fig. 2b). Prior to the pBoc (t = −5…0 sec), a rise in anterior curvature coincided with the transient decline in the propensity for backward motion. At the time of the pBoc, the animal relaxed its curvature at both the anterior and the posterior of the body. Posterior curvature then rapidly increased, peaking between the two Bocs while anterior curvature remained suppressed. During the aBoc, anterior curvature rose (and peaked shortly thereafter) while posterior curvature was suppressed. Therefore, both locomotion and posture are modulated in concert with the DMP cycle. We note that these stereotypical dynamics were observed in each and every of the wild-type animals assayed (as reflected in the low animal to animal variability in Fig. 2).

### The DMP and the DAMP co-terminate and co-reset

**Figure 3:**
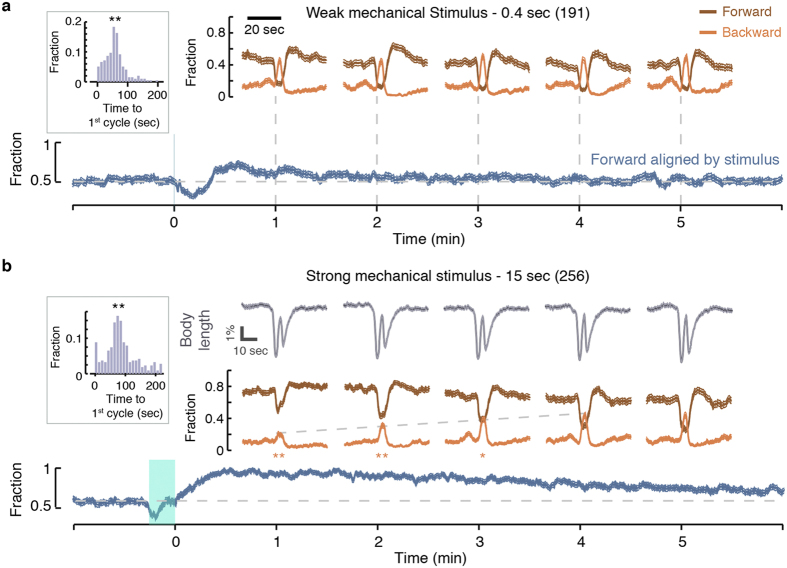
The DMP and the DAMP co-reset. (**a**) Top: the propensities for forward and backward locomotion of wild-type animals during the first five DMP cycles following a 0.4 sec mechanical stimulus that terminated at t = 0. Bottom: the propensity for forward locomotion aligned at the onset of the stimulus. The brief stimulus evoked a mild forward response. Data shown here was reproduced from^71^. Top (averaged by Boc) and bottom (averaged by stimulus) traces were plotted with the same time scale and each top 50 sec epoch was positioned at approximately its correct location along the continuous time axis. Inset: a histogram of the delays of the first DMP cycle following the mechanical stimulus. As previously reported^12^, the stimulus resets the pacemaker such that most cycles initiate around at t = 60 sec. (**b**) Same as (**a**), but for a 15 sec mechanical stimulus that terminated at t = 0 and evoked a strong forward response. The longer stimulus reset the DMP pacemaker and did not detectably affect DMP body contractions (grey traces). DAMP cycles remained synchronized with the DMP, but the amplitude of the backward motion peaks was inversely correlated with the overall propensity for forward locomotion. The numbers of stimuli assayed are depicted in parentheses. Thin lines depict mean ± s.e.m. Double asterisks depict significant differences from a histogram of first cycle times after a mock stimulus or significant differences in peak propensities for backward motion as compared to the fifth cycle after the stimulus (p < 0.01).

Gentle touch near the head resets the defecation cycle pacemaker – it delays subsequent defecation by one normal period regardless of when the stimulus was delivered^12^. Similarly, we were able to reset the defecation clock using short (0.4 sec) or long (15 sec) vibration stimuli at a frequency of 1 kHz, administered at random phases with respect to the DMP cycle (see insets in Fig. 3a,b).

To test whether the DAMP co-reset with the DMP, we separately assayed each of the first five cycles after the short mechanical stimulus (Fig. 3a). The two motor programs could have been temporarily dephased if the DAMP was not co-reset with the DMP. In that case, DAMP dynamic would have averaged out when aligned by the first post-stimulus pBoc. This was not the case: the DAMP was readily detectable during the first post-stimulus DMP cycle, indicating that the two motor programs co-reset.

Directional locomotion of *C. elegans* is established by an imbalance between the outputs of the forward and backward motor circuits. Altering the direction of locomotion is achieved by switching between the forward-favoring and backward-favoring imbalanced states^34^. Would circumstances under which forward locomotion is more favorable impede this switch and suppress DAMP backward motion? To address this question we delivered long mechanical stimuli (Fig. 3b).

Briefly after the stimulus, the probability of forward locomotion neared unity. Over the next several minutes, it declined linearly to its baseline value. Thus, the stimulus generated a gradient in the probability to locomote forward that spanned several DMP cycles. The stimulus did not affect DMP body contractions nor did it affect the synchrony between the DMP and the DAMP. However, the amplitudes of the DAMP backward motion peaks were inversely related to the forward favoring imbalance, i.e., switching to backward motion was suppressed in a forward probability dependent manner (Fig. 3b). A similar trend was observed using blue light stimuli, which reset the DMP pacemaker by either inducing a cycle at the time of the stimulus or delaying the cycle by a single period (Fig. S3).

We further asked if the DAMP co-terminated with the DMP. To address this, we assayed animals during 10-minute intervals leading to and immediately after the onset of L4 lethargus, a stage in which feeding and defecation are suspended^42^. During the first 10 min of L4 lethargus the DMP Bocs persisted and the corresponding DAMP dynamics of body posture were detected (Figs S4a). At this time, DAMP modulation of backward motion was weak but detectable and forward locomotion was rare and uncorrelated with the Bocs. The reduced coupling between body curvature and directed locomotion is consistent with previous observations of the loss of synchrony between backward interneurons^43^ and the near elimination of directed locomotion during lethargus^40^.

Between 10 and 20 minutes after the onset of lethargus, detected body contractions were predominantly associated with transitions between quiescence and motion (see below), as defecation cycles ceased. Correspondingly, both posture and locomotion reflected the patterns associated with these transitions, rather than DAMP-like dynamics. Thus, the DMP and DAMP remained tightly coupled in our assays, i.e., they co-reset instantly and co-terminated during lethargus.

We note that during lethargus small non-periodic modulations of body length were readily detected. Locomotion behavior during this stage is composed of alternating bouts of quiescence, when body-wall muscles are typically relaxed^44^,^45^, and bouts of motion^46^,^47^. Aligning the body length data at the onset of quiescence bouts revealed a 5 sec phase of rapid relaxation in preparation for quiescence, followed by a more gradual elongation of the body throughout the bout. Motion bouts were initiated with a 4 sec phase of rapid contraction and ended in the relaxation prior to the next quiescence bout (Fig S4b). These dynamics provide threshold-free distinct physiological correlates to the two types of bouts. Thus, they support the idea that motion and quiescence during lethargus are distinct physiological micro-states.

### Intestinal calcium waves, not pBocs, correlate with the DAMP peak in backward motion

How are the DMP and the DAMP coupled? The symmetric contraction of posterior body muscles (pBoc) relaxes asymmetrically with respect to the dorsal/ventral directions, resulting in a posterior body-bend. This is evident in the peak in posterior curvature that is established as the pBoc relax (Fig. 2b) and readily confirmed upon visual inspection (movie M1). In a simple model, this posterior bend would trigger the propagation of dorsoventral bends towards the anterior, perhaps through proprioceptive coupling^48^. If this were the case, the execution of a pBoc would be essential for initiating DAMP backward motion. To test this model, we assayed animals lacking the function of genes known to be required for pBocs.

**Figure 4:**
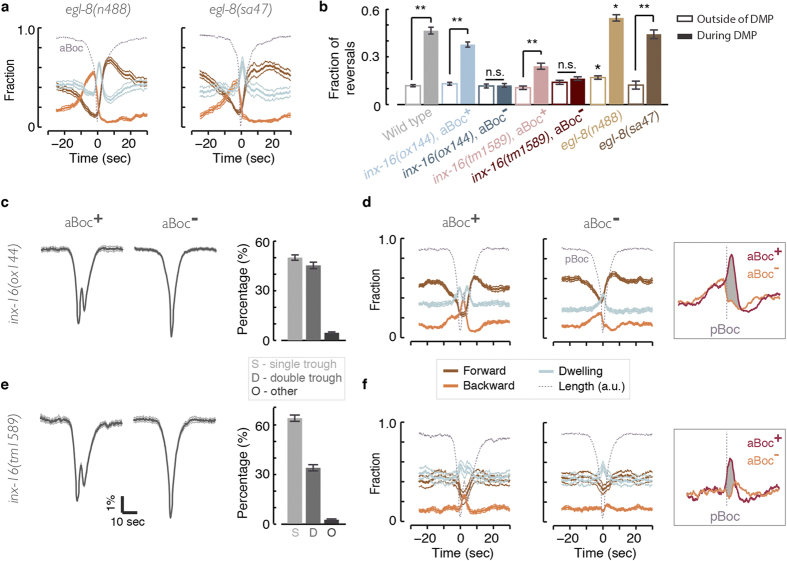
Intestinal calcium waves, but not pBocs, drive the DAMP peak of backward motion. (**a**) The likelihood of observing forward motion, backward motion, and dwelling during DMP cycles with no detectable pBocs in *egl-8(n488)* and *egl-8(sa47)* mutants. Note that here t = 0 was defined at the trough of the aBoc. (**b**) Baseline propensities (empty bars, measured 20–25 sec prior to the pBoc or 20–25 sec prior to the aBoc) and peak DAMP propensities (solid bars) for backward motion of wild-type animals (data reproduced from Fig. 4 for convenience), *egl-8* mutants, and *inx-16* mutants. Data from *inx-16* mutants was separated to two groups based on whether an aBoc was detected. Error bars denote denote ± s.e.m, N(*n488*) = 9, N(*sa47*) = 4, N(*ox144*) = 13, and N(*tm1589*) = 8 animals, Double asterisks denote significant differences (p < 0.01). Single asterisks above *egl-8(n488)* bars denote differences from corresponding wild-type data (p < 0.05). (**c**) Left: the mean body length of *inx-16(ox144)* mutants during single- and double-trough events. Right: the fractions of detected single, double, and multiple body-length troughs during the L4 stage of *inx-16(ox144)* mutants. (**d**) Same as panel (**a**) for cycles of *inx-16(ox144)* mutants where an aBoc was detected (left) or not detected (right). Inset: the post-pBoc phenotype of *inx-16(ox144)* mutants is denoted by the shaded area between the overlaid traces of backward motion propensities in the presence and absence of the aBoc. (**e**,**f**) Same as panels (**c**,**d**) for *inx-16(tm1589)* mutants. In all panels, dotted grey lines represent body-length in arbitrary units as a guide to the eye, thin lines and error bars denote mean ± s.e.m.

The function of the *C. elegans* phospholipase C (PLC)-β homologue, EGL-8, is known to be required for pBocs^12^. Its effect on intestinal calcium dynamics and the execution of the DMP were most closely examined using the *egl-8(n488)* loss-of-function allele. In *egl-8(n488)* mutants, intestinal calcium waves are not eliminated but initiate ectopically and arrhythmically, often failing to trigger a pBoc^26^,^49^. Nevertheless, *egl-8(n488)* mutants exhibited an elevated propensity for DAMP backward motion even in the complete absence of a pBoc, as well as an increase in their baseline propensity to move backwards (Fig. 4a,b). The onset of the elevation in *egl-8(n488)* backward motion occurred 15 sec prior to the aBoc, i.e., at the same time that it was detected in wild-type animals with respect to the DMP cycle. Animals carrying an additional loss of function alleles*, egl-8(sa47)*^12^, exhibited a similar phenotype (Fig. 4a,b). Therefore, the execution of a pBoc in and of itself is not required for promoting backward motion in phase with the DMP cycle.

Next, we asked whether the calcium waves, typically originating at the posterior intestine and traveling towards the anterior, instruct backward locomotion during the DAMP. The pannexin intestinal gap-junction unit, INX-16, propagates the intestinal calcium waves^24^. Importantly, intestinal calcium dynamics and body contractions were co-analyzed in *inx-16(ox144)* mutants, where calcium waves were either eliminated, misdirected, or merely retarded. This analysis identified a one-to-one correlation between the position (i.e., intestinal ring), direction, and speed of the calcium wave and body-contractions. In addition, propagation of the calcium wave to the anterior intestine was abolished or severely defective in *inx-16(ox144)* mutants. Correspondingly, the majority of *inx-16* aBocs are absent, since calcium flux in the anterior is required to trigger the aBoc and Exp steps^23^,^24^,^26^. Thus, intestinal calcium was shown to instruct the DMP body contractions.

To address whether intestinal calcium waves also instruct DAMP backward motion we separated the cycles of *inx-16(ox144)* mutants to two groups based on whether an aBoc was detected or not (Fig. 4c,d). The former group was guaranteed to be less defective in the propagation of the calcium wave to the anterior, as this was required for aBocs. Strikingly, the two groups displayed a robust phenotypic difference in every animal that was assayed. The post-pBoc peak indicating upregulation of backward motion was clearly present when an aBoc was detected. This peak was completely absent otherwise, i.e., none of the animals upregulated backward motion when aBocs were not detected (Fig. 4b–e). In contrast, outside of the epoch between the Bocs backward motion in these two cases was indistinguishable, including an identical pre-pBoc rise in the propensity to move backward.

To confirm that the observed phenotype was caused by the loss of function of INX-16 we assayed a second allele, *inx-16(tm1589)*^24^. These mutants were visibly more constipated than *inx-16(ox144)* animals, suggesting a stronger deficiency in the function of the gene. Correspondingly, we could detect pairs of body contractions only in 30% (as opposed to 45%) of the DMP cycles (Fig. 4c,e) and the DAMP peak of backward motion was smaller. Nevertheless, when cycles were separated to two groups based on the detection of aBocs, the striking phenotype was clearly exhibited by *inx-16(tm1589)* mutants as well (Fig 4d,f).

Several studies examined the expression pattern of *inx-16* using transcriptional and translational reporters and found robust expression exclusively in the intestine^24^,^50^,^51^. Combined, our data support the notion that intestinal calcium flux is required for the post-pBoc enhancement of backward motion and are inconsistent with the simple model where a successful execution of the pBoc is required for triggering backward motion during the DMP cycle. Under this interpretation, the data implicate the intestine in affecting the imbalance between backward and forward locomotion. However, we did not rule out the possibility that an unknown upstream factor, which may be is inconsistently active in *inx-16* mutants, drives both intestinal calcium and backward locomotion independently.

### Proton signaling plays a role in modulating backward motion during the DMP cycle

Intestinal calcium waves were shown to drive proton signaling, required for execution of pBocs, from the intestine to the overlying muscles: the PBO-4 Na^+^/H^+^ exchanger releases protons from intestinal cells across the basolateral membrane, where they activate the proton-gated cation channel PBO-5/PBO-6 in the posterior muscles to stimulate the posterior contraction^25^,^27^. To address whether proton signaling was also required to relay the intestinal calcium signal to the locomotor circuit we examined the effect of the loss of function of PBO-4/5 on the DAMP.

**Figure 5:**
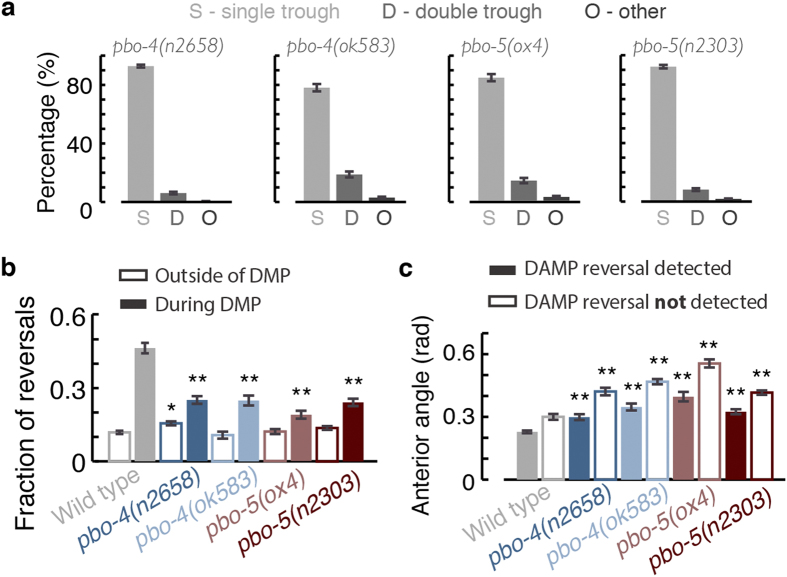
Proton signaling, but not the execution of the pBoc in and of itself, promotes backward motion during the DMP cycle. (**a**) The fractions of detected single, double, and multiple body-length troughs during the L4 stage of *pbo-4* and *pbo-5* pBoc deficient mutants. (**b**) Baseline propensities (empty bars, measured 20–25 sec prior to the aBoc) and peak DAMP propensities (solid bars) for backward motion of wild-type (data reproduced from Fig. 4), pbo-4, and *pbo-5* mutants. (**c**) The absolute anterior angles during DMP cycles in which backward motion was detected (solid bars) or not detected (empty bars) for wild-type animals (data reproduced from Fig. 4), *pbo-4*, and *pbo-5* mutants. N(wild-type) = 28, N(*pbo-4(n2658)*) = 11, N(*pbo-4(ok583)*) = 13, N(*pbo-5(ox4)*) = 13, and N(*pbo-5(n2303)*) = 10. Error bars depict ± s.e.m. Single and double asterisks denote significant differences from wild-type (p < 0.05 and p < 0.01, respectively).

pBocs were eliminated in *pbo-4* and *pbo-5* mutants assayed in our machine vision system (Fig. 5a). As confirmed by visual inspection, the small fraction of double contractions was mainly due to experimental noise and detection errors. Both *pbo-4* and *pbo-5* mutants exhibited wild-type propensities for backward motion outside of the DAMP and a significant reduction in DAMP backward motion (Fig. 5b). However, some elevation of backward motion during DMP cycle was still detectable in both of these mutants.

Wild-type animals suppress anterior curvature between the Bocs (Fig. 2b) and preferentially do so when backward locomotion is detected at that time (Fig. 5c). Although *pbo-4* and *pbo-5* mutants exhibited elevated higher anterior curvature than wild-type, they still exhibited preferential anterior straightening concurrent with DAMP backward motion (Fig. 5c). Qualitatively, the posture and locomotion dynamics of *pbo-4* mutants during the DMP cycle retained many of the trends observed in wild-type animals (Fig. 5b,c and S5). Thus, proton signaling promoted, but was not strictly required, for the DAMP. Taken together, our genetic data indicate that pBocs are not required for increased backward motion prior to expulsion, since the two are genetically separable, and that proton release driven by the intestinal calcium wave contributes to the coupling between the DMP and the DAMP.

### Vesicle exocytosis from AVA or AVE does not strongly affect the modulation of backward motion during the DMP cycle

**Figure 6:**
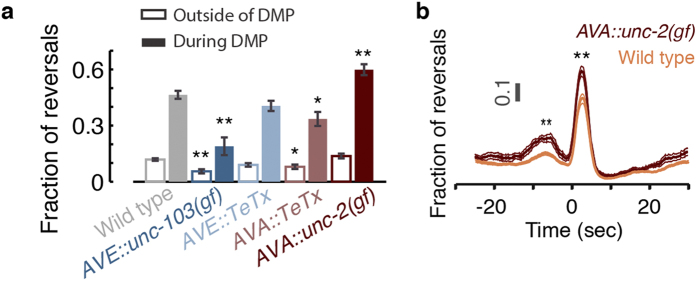
Vesicle exocytosis from AVA and AVE is not essential for the DAMP peak in backward motion. (**a**) Baseline propensities (empty bars, measured 20–25 sec prior to the pBoc) and peak DAMP propensities (solid bars) for backward motion of wild-type and four transgenic strains with altered AVA or AVE activity. (**b**) The temporal dynamics of backward motion of *AVA::unc-2(gf)* transgenics. Both the minor (t = −8 sec) and the major (t = 2 sec) backward motion peaks are enhanced as compared to wild-type. Error bars or thin lines depict ± s.e.m. N(wild-type) = 28, N(*AVE::unc-103(gf)*) = 11, N(*AVE::TeTx*) = 11, N(*AVA::TeTx*) = 9, and N(*AVA::unc-2(gf)*) = 10 animals. Single and double asterisks denote significant differences from wild-type (p < 0.05 and p < 0.01, respectively).

To test the roles of the motor circuit neurons AVA and AVE in promoting backward locomotion between DMP body contractions, we used the tetanus toxin light chain (TeTx) for blocking vesicle secretion and a gain of function allele of the *unc-103* potassium channel for hyperpolarizing neurons^34^,^52^,^53^. If vesicle exocytosis output from AVA or AVE is required, genetically silencing them should suppress the observed backward motion peak. However, the peak fraction of DAMP backward motion was unaffected or only mildly reduced after expressing TeTx in AVE or AVA, respectively (Fig. 6a). Hyperpolarizing AVE resulted in a substantial reduction of backward motion during the DMP cycle and increased the baseline probability to locomote forward. Premotor interneurons were previously implicated in modulating motoneuron activity to establish directionality of movements through gap junctions^34^. The difference between the phenotypes of *TeTx*- and *unc-103(gf)*-expressing transgenics can be explained in light of these findings.

Since the propensity for backward motion during the DAMP was mildly reduced in AVA::TeTx transgenics, hyperactivation of AVA would be expected to result in an opposite phenotype. Therefore, we expressed a gain-of-function (gf) allele of the *unc-2/CaV2* gene in the AVA neurons. UNC-2/CaV2 is the α-subunit of a P/Q/N-type voltage-gated calcium channel (Ca_v_2) which mediates Ca^2+^ influx that triggers neurotransmitter release. The *unc-2/CaV2(gf)* mutation produces a negative shift in channel activation and significantly increases Ca^2+^ influx (YC and MA, in preparation). Cell specific expression of the *unc-2/CaV2(gf)* allele in AVA neurons (*Prig-3::UNC-2GF*) resulted in increasing the fraction of backward motion during the DMP cycle without significantly affecting the baseline propensity to move backward (Fig. 6a). The pre-pBoc elevation in backward motion was also enhanced in *Prig-3::UNC-2GF* transgenics (Fig. 6b). This further indicated that the onset of the DAMP occurred 10 seconds before the pBoc and preceded the reported calcium transient in the posterior intestine. Together, these findings suggest that vesicle secretion from AVA contributes to, but is not essential for DAMP backward motion.

We note that since aBocs and expulsions take place at opposite ends of the animal, scoring them concurrently by visual inspection is challenging and, in practice, avoided. Although aBocs^36^ and expulsions^20^,^31^ were associated with peptidergic signaling in separate studies, the question of whether they are mechanistically separable was not addressed. We observed severe aBoc defects in *unc-31*, *egl-3*, and *egl-21* mutants and in *Popt-3::TeTx* and *Prig-3::TeTx* transgenics (Figs S1 and S6). However, only *egl-21* mutants and the transgenics exhibited expulsion defects and appeared constipated, i.e., neither *unc-31* nor *egl-3* mutants exhibited these phenotypes (Fig. S6e). Therefore, aBocs and expulsions are separable.

### aBocs and proton signaling are required for modulating backward motion during the DAMP

The propensity to move backward during the DAMP is characterized by two peaks: a small peak prior to the pBoc and a more prominent one between the Bocs (Figs 2a and 6b). Therefore, backward motion is suppressed twice: just before (or during) the pBoc and during the aBoc. The execution of a body contraction in and of itself may be sufficient to terminate backward motion. If so, the absence of that Boc would always result in extending backward motion as compared to wild-type. Alternatively, if independent signals modulate backward motion then the patterns of DAMP locomotion may be preserved even in the absence of a body contraction. To address this, we examined more closely the patterns of backward motion in aBoc and pBoc deficient mutants.

**Figure 7:**
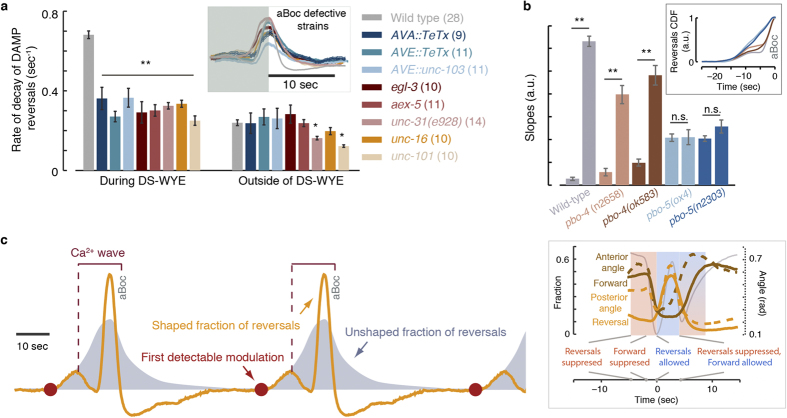
aBocs and proton signaling are required for shaping the DAMP locomotion dynamics. (**a**) Rates of decay of backward motion during the DAMP and outside of the DAMP for wild-type animals and aBoc deficient mutants. Inset: the temporal dynamics of pBoc aligned backward motion. (**b**) The slopes obtained from fitting a piecewise linear function to the scaled cumulative propensities of backward motion of pBoc deficient mutants. For each strain, the left and right bars correspond to the slope at t = −15..−5 sec and t = −5..0 sec prior to the aBoc, respectively (see also Fig. S7). The numbers of animals in each dataset are denoted in parentheses. Inset: the scaled cumulative propensities of backward motion are shown for the purpose of demonstration. Double asterisks in panels (a, b) denote a significant difference (p < 0.01). (**c**) A graphical representation of a model. The first detectable modulation of locomotion and the basal elevation in the propensity of backward motion are denoted by red circles and shaded areas, respectively. The intestinal calcium wave originates at the posterior just prior to the pBoc (vertical dotted line) and terminates at the anterior just prior to the aBoc. This period is denoted by the horizontal bracket. The observed dynamics of backward motion (orange curve) are shaped by additional signals, including proton signaling driven by the calcium wave between the Bocs. Inset: The mean wild-type dynamics of locomotion and body curvature overlaid on a single detailed timeline.

To ask whether an aBoc terminated backward motion in and of itself we aligned locomotion dynamics from individual DMP cycles at the onset of backward motion after the pBoc and measured the resulting decay rate. In all strains with aBoc defects, these rates were nearly 2-fold slower than wild-type, and similar to the relaxation rates of spontaneous reversals outside of the DMP cycle (Fig. 7a). Thus, we were not able to separate aBocs from the timely termination of DAMP backward motion.

To ask whether a pBoc suppressed backward motion in and of itself, we examined the patterns of locomotion in *pbo-4* and *pbo-5* pBoc deficient mutants. The absence of a pBoc in *pbo-4* mutants did not abolish the suppression of backward motion 10 seconds prior to the aBoc, i.e., 5 seconds prior to the calculated time of the missing pBoc. In contrast, the propensity of *pbo-5* mutants to move backward increased gradually and monotonically during the 15 sec prior to the aBoc (Figs S5 and S7). To quantify the difference in the functional form of the propensities to move backward, we scaled the cumulative propensities at t = −25…0 sec before the aBoc such that their values were between 0 and 1. The scaled data was fit to a piecewise linear function (Fig. 7b inset and Fig. S7) and the slopes between t = −15..−5 sec and t = −5..0 with respect to the aBoc were quantified.

Shaping of backward motion dynamics persisted despite the loss of function of PBO-4, as evident by the 5-fold difference between the two slopes. In contrast, the two slopes were indistinguishable in *pbo-5* mutants (Fig. 7b). Residual oscillations of pseudocoelomic pH and a capability of rhythmic posterior alkalization in intestinal cells were previously observed in *pbo-4* mutants. These could result from the action of additional exchangers in the intestinal basolateral membrane^21^,^26^,^27^. Thus, consistent with their residual DMP phenotypes, *pbo-4* mutants exhibited a DAMP defect that was less severe. These findings indicated that activating the proton-gated cation channel, but not the pBoc in and of itself, was required for shaping DAMP locomotion dynamics leading to the time of the pBoc.

## Discussion

*C. elegans* motor programs include locomotion, escape responses, egg laying, male mating, and defecation^54^. Escape responses and male mating, for instance, require coordinated sequences of discernible steps^55^,^56^,^57^,^58^,^59^. However, modulation of the speed of locomotion in coordination with egg-laying was the only reported example of coordination between seemingly distinct motor programs^60^. The coordination between the DMP and the locomotion sequence identified as the DAMP provides the first example in *C. elegans* of two multi-step motor programs being executed concurrently and in synchrony. Yet, the two programs are separable as wild type animals can execute the former without fully executing the latter.

What might be the utility of the DAMP? Under optimal laboratory conditions, where *C. elegans* is cultivated on an effectively infinite and uniform food covered surface, brief reversals would presumably not have a significant impact. However, when food is not distributed uniformly, food sources can be contaminated or degraded in quality. Complex (e.g., patchy) spatial distributions of resources arise naturally due to the interactions of multiple species and environmental factors^61^,^62^,^63^. Moreover, heterogeneity in foraging systems is known to affect locomotion-based behaviors such as foraging or patch leaving^2^,^64^,^65^,^66^,^67^.

When resources are localized to small patches the trade-offs of performing an avoidance-like behavior may become more favorable than in the optimal laboratory case. One possible advantage of coupling locomotion to defecation could thus be the reduction of parasite transmission and infection, which would affect population dynamics^2^,^7^,^64^. Indeed, retaining pathogenic bacteria in the intestine severely affects the survival of *C. elegans* and *P. pacificus*, as defecation mutants are hyper-susceptible to pathogens^68^. Another, not mutually exclusive, advantage of the DAMP may be the enhancement of the quality of consumed food in a complex foraging environment.

A key component of DAMP is the stereotypical dynamics of backward locomotion: (i) an unidentified cue triggered backward motion 10 seconds prior to the pBoc; (ii) proton signaling was required for shaping the observed locomotion patterns; (iii) intestinal calcium instructed the peak in backward motion between the Bocs; and (iv) the execution of the aBoc suppressed backward motion at the end of the DMP cycle. Intestinal calcium waves in *egl-8* mutants were shown to be highly arrhythmic, to sometimes initiate in the middle of the intestine and/or propagate rearward, and to propagate at abnormally slow speeds^26^,^49^. The loss of the stringent temporal control of DAMP backward motion in *egl-8* mutants is consistent with such slow and aberrant calcium dynamics, in support the notion that intestinal calcium can drive backward locomotion.

It is unknown how the synchrony between the DMP and the DAMP, summarized in the timeline depicted in Fig. 7c (right), is preserved. A plausible hypothesis, compatible with our data, is that a common upstream cue triggers two events: (i) titling of the balance between the forward and backward motor circuits in favor of the latter^34^; and (ii) physiological dynamics in the intestine leading to the initiation of the DMP^24^,^25^,^26^. Since the distribution of periods between consecutive DMP cycles has a non-negligible width (see, e.g., Fig. 1d), relaxing the assumption of a common trigger would necessitate a mechanistic explanation for the two motor programs remaining in phase.

In summary, we presented a method that enabled us to concurrently assay the defecation ultradian rhythm and locomotion in *C. elegans*. Our data demonstrated a previously unnoticed coupling between the two. Interestingly, a simple model for implementing the coupling between the DMP and the DAMP, requiring merely proprioceptive coupling similar to that previously described^48^, was not supported by the data. Instead, intestinal calcium wave instructs directed locomotion between the Bocs and proton signaling pays a role in this process.

## Materials and Methods

### Strains

*C. elegans* strains were maintained and grown according to standard protocols^69^. The following strains were used: wild-type strain N2, CB169 *unc-31(e169)*, CB928 *unc-31(e928)*, MT1541 *egl-3(n729)*, MT1241 *egl-21(n611)*, MT1542 *unc-16(n730)*, JT23 *aex-5(sa23)*, KG532 *kin-2(ce179)*, KG518 *acy-1gf(ce2)*, KP1182 *acy-1(nu329)*, QW89 *lgc-55(tm2913)*, QW284 *tdc-1(n3420)*, MT1083 *egl-8(n488)*, JT47 *egl-8(sa47)*, EG144 *inx-16(ox144)*, EG3233 *inx-16(tm1589)*, EG1389 *pbo-4(n2658)*, RB793 *pbo-4(ok583)*, EG4 *pbo-5(ox4)*, *pbo-5(n2303)*, OS4976 nsEx2846[*popt-3::TeTx*, *elt-2::mCherry*], OS6515 nsEx3649[*popt-3::unc103(gf)*], OS6517 nsEx3651[*popt-3::unc103*(gf)], QW714 *lin-15(n765ts)*; zfEx286[*prig-3::unc-2(gf)*], INV30015 somEx[*prig-3::TeTx::SL2::mCherry*], INV30016 somEx[*prig-3::TeTx::SL2::mCherry*].

### Locomotion Assays

Animals were grown at 20 °C on standard NGM plates seeded with *E. coli* OP50 bacteria. Mid to late L4 individuals were sealed into individual “artificial dirt” chambers filled with an overnight OP50 culture concentrated tenfold and resuspended in NGM medium^70^. Animals were imaged at 10 frames per second at a 4.2× magnification for posture-based analysis using a CCD camera (Prosilica GC2450, Allied Vision Technologies, Stadtroda, Germany). Motion and quiescence were identified using previously described methods^41^.

### Expulsion Assays

First day young adult animals are picked onto a freshly seeded plate. After a 15–20 minute period of acclimation, 10 consecutive DMP cycles are manually scored for successful expulsions as previously described^12^,^37^. The success rate is calculated as a ratio of expulsions to posterior contractions.

### External stimuli

Mechanical stimuli were generated using 50 mm piezo buzzer elements (Digikey part no. 668–1190-ND) as previously described^41^. The timing and duration of the stimuli were controlled using a custom Matlab script. Blue light (λ = 475 ± 15 nm) was supplied by a Luxeon Star 7-LED assembly with a diffused optic array driven by a 700 mA FlexBlock driver^71^. The timing of light stimuli was controlled using LabView (National Instruments Inc., Austin TX). All stimuli were delivered repeatedly at 15 min intervals to L4 larvae.

### Analysis of behavior

Precision measurements of body length were performed as previously described using a custom suite of tools, called PyCelegans^40^,^41^. In brief, we identified the body midline in each frame, as well as the positions of the head and the tail. Each midline was divided into 20 equal intervals and the dynamics of the angles between these intervals were used to identify quiescence and directed locomotion states. The onset of lethargus was identified by visual inspection of quiescence data. The troughs in body length that resulted from pBocs and aBocs were identified using custom Matlab scripts (Mathworks Inc., Natick MA). The raw, 10 fps, data was smoothed using a 2 sec window to eliminate high frequency measurement noise and using a 100 sec window to generate a baseline. The smoothed data was normalized by the baseline. We found that calculating the mean body length repeatedly for each 10-minute segment of the experiment resulted in reliable threshold-based trough detection (where the threshold was set to one standard deviation below the mean). A temporal window of 10 sec was used to classify contraction events as ‘single trough’, ‘pair of troughs’, or ‘multiple troughs’. Visual inspection indicated that single and multiple trough events could result from measurement error in wild-type animals. DMP contractions and DAMP locomotion patterns were also confirmed by visual inspection of freely behaving animals on standard cultivation plates. Locomotion dynamics and in particular the frequency of DAMP backward locomotion was similar on plates to the data obtained from our automatic analysis for all of the strains assayed.

### Statistical and Numerical Analysis

Data analysis was performed using custom Matlab scripts, using the statistics and curve fitting toolboxes. Summary statistics for locomotion behavior were calculated using 5 and 3 sec windows for baseline and peak values, respectively. Summary statistics for the anterior angle (used to assess head swings) were calculated using 5 and 8 sec windows for baseline and DMP cycle values, respectively. For comparisons in summary statistics panels, significance was calculated using a one-way ANOVA test. Post-hoc correction for multiple comparisons was performed using the Bonferroni adjustment. The Kruskal–Wallis test was used to show that the histograms in Fig. 3 did not originate from the same distribution as a mock stimulus histogram.

## Additional Information

**How to cite this article**: Nagy, S. *et al*. *Caenorhabditis elegans* exhibit a coupling between the defecation motor program and directed locomotion. *Sci. Rep*. **5**, 17174; doi: 10.1038/srep17174 (2015).

## Supplementary Material

Supplementary Information

Supplementary Movie S1
