# Supplementary material for: Caenorhabditis elegans exhibit a coupling between the defecation motor program and directed locomotion
Source: Sci Rep. 2015 Nov 24;5:17174. doi: 10.1038/srep17174 (PMC4657007; doi:10.1038/srep17174)
Supplement: Supplementary Information [file srep17174-s1.pdf]

Caenorhabditis elegans exhibit a coupling between the defecation motor program and directed locomotion.

Stanislav Nagy[1], Yung-Chi Huang[2], Mark J. Alkema[2], and David Biron[1,3]

1. The Institute for Biophysical Dynamics, The University of Chicago, Chicago, IL

2. Department of Neurobiology, University of Ma ssachusetts Medical School, Worcester, MA

3. Department of Physics and the James Franck Institute, The University of Chicago, Chicago, IL

SUPPLEMENTARY FIGURES AND FIGURE LEGENDS

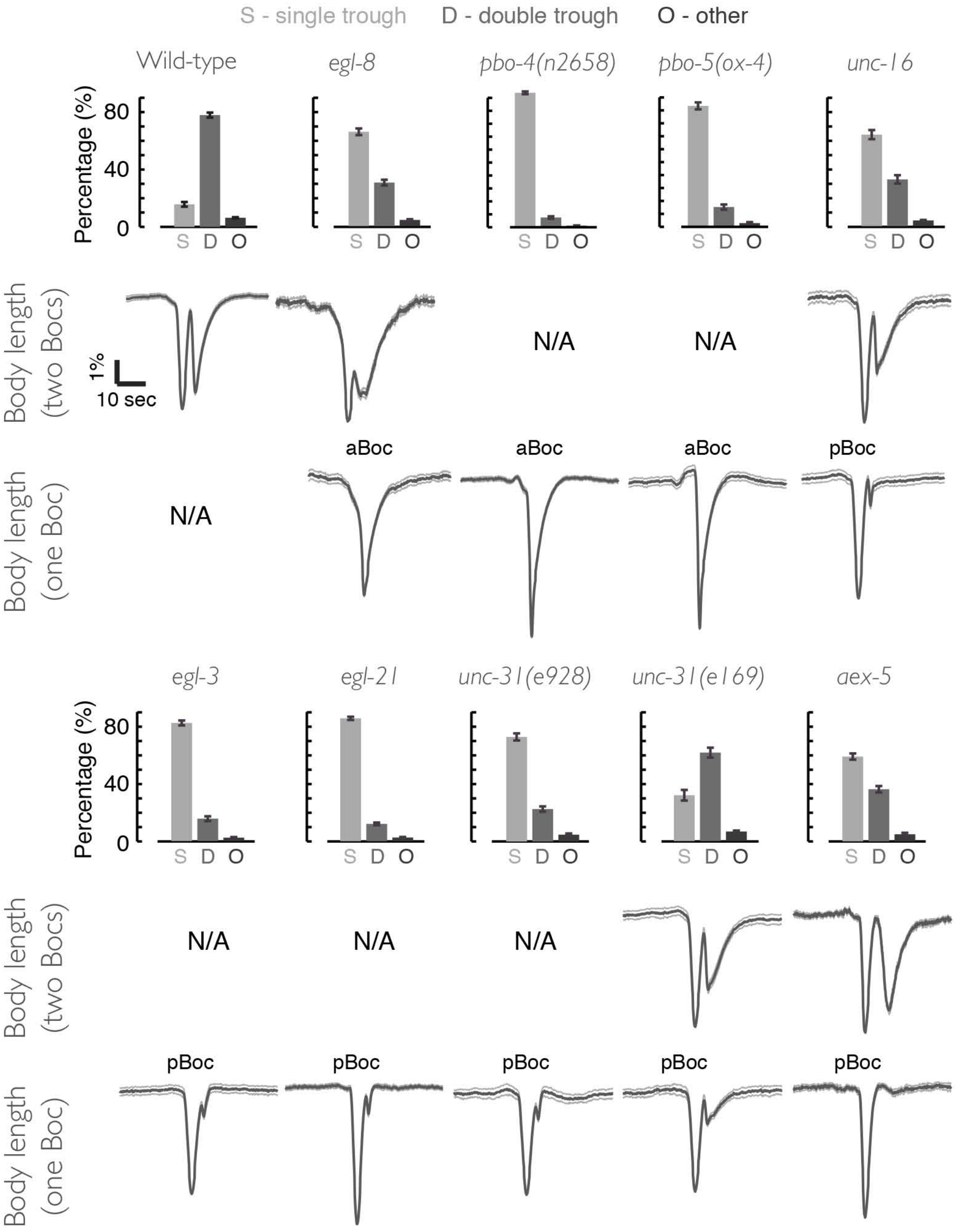

S - single trough
D - double trough
O - other
Wild-type
egl-8
pbo-4(n2658)
pbo-5(ox-4)
unc-16
Percentage (%)
80
40
0
S D O
Body length (two Bocs)
Body length (one Boc)
1%
10 sec
N/A
aBoc
pBoc
egl-3
egl-21
unc-31(e928)
unc-31(e169)
aex-5

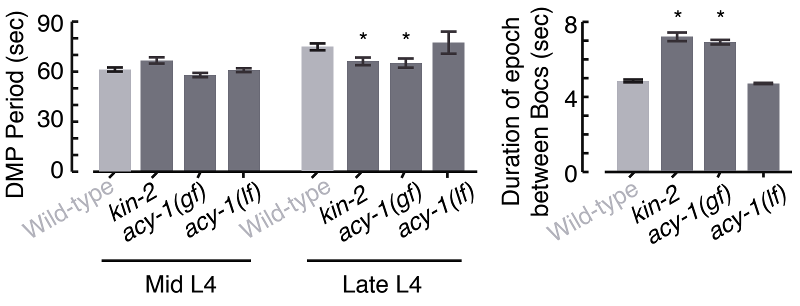

DMP Period (sec)
90
60
30
0
Wild-type
kin-2
acy-1(gf)
acy-1(lf)
Wild-type
kin-2
acy-1(gf)
acy-1(lf)
Mid L4
Late L4
*
*
Duration of epoch between Bocs (sec)
8
4
0
Wild-type
kin-2
acy-1(gf)
acy-1(lf)
*
*

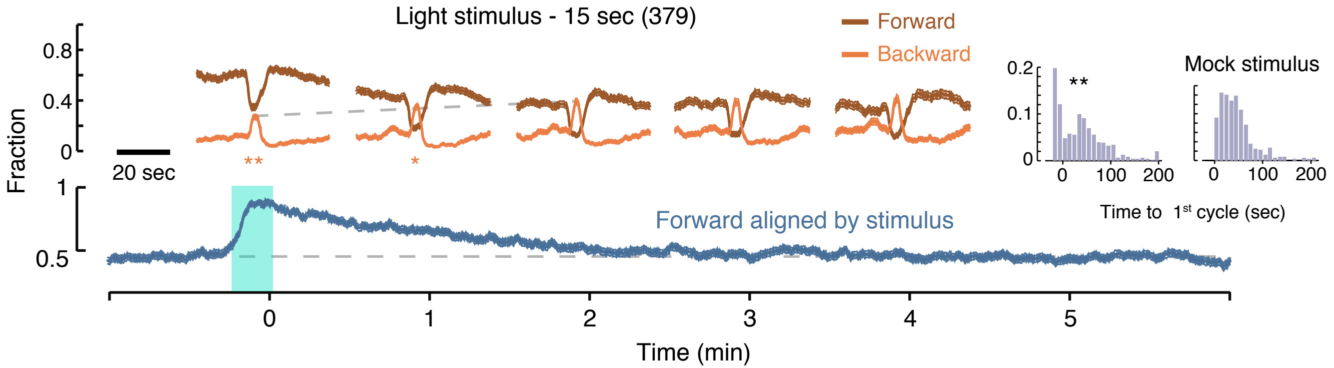

Light stimulus - 15 sec (379)
Forward
Backward
Fraction
0.8
0.4
0
20 sec
**
*
1
0.5
Forward aligned by stimulus
0.2
0.1
0
**
0
100
200
Mock stimulus
0
100
200
Time to 1st cycle (sec)
0
1
2
3
4
5
Time (min)

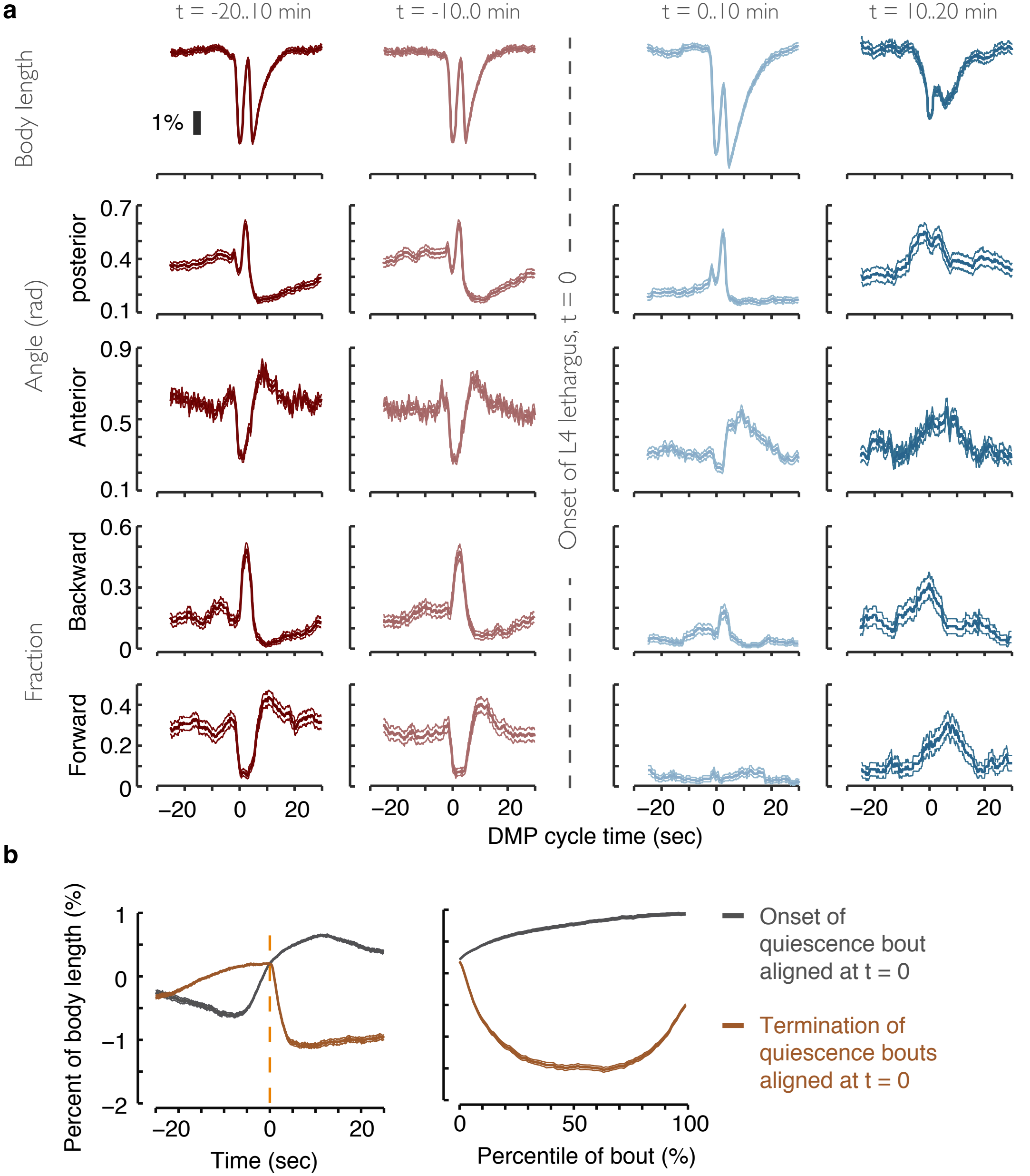

a
t = -20..10 min
t = -10..0 min
t = 0..10 min
t = 10..20 min
Body length
1%
Angle (rad)
posterior
Anterior
Fraction
Backward
Forward
0.7
0.4
0.1
0.9
0.5
0.1
0.6
0.3
0
0.4
0.2
0
Onset of L4 lethargus, t = 0
–20
0
20
DMP cycle time (sec)
b
Percent of body length (%)
1
0
–1
–2
Time (sec)
Percentile of bout (%)
0
50
100
Onset of quiescence bout aligned at t = 0
Termination of quiescence bouts aligned at t = 0

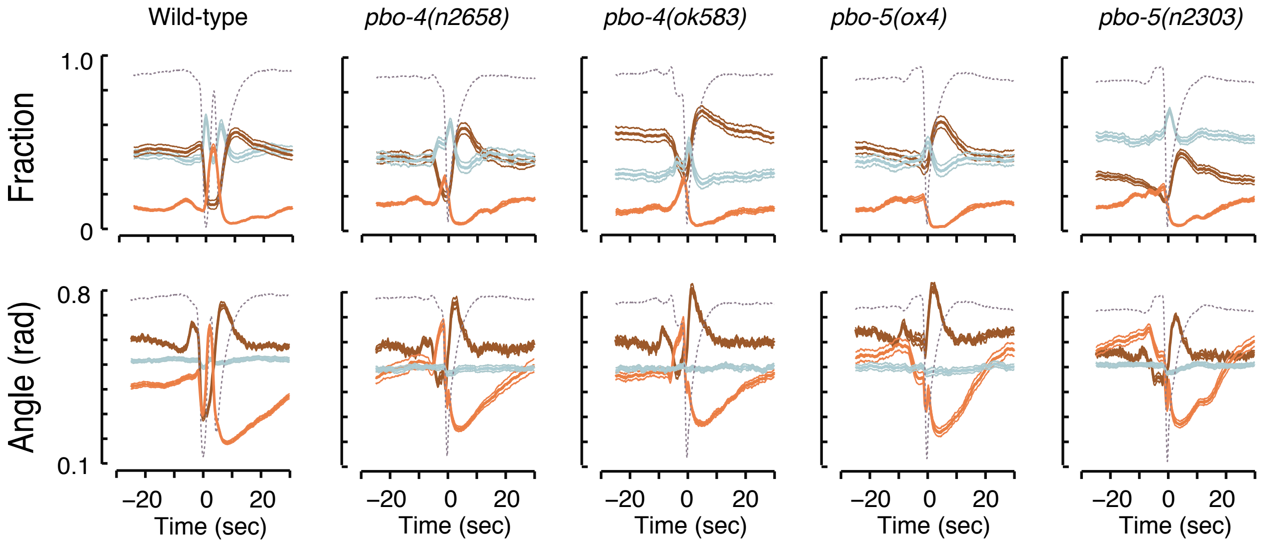

Wild-type
pbo-4(n2658)
pbo-4(ok583)
pbo-5(ox4)
pbo-5(n2303)
Fraction
1.0
0
Angle (rad)
0.8
0.1
−20
0
20
Time (sec)

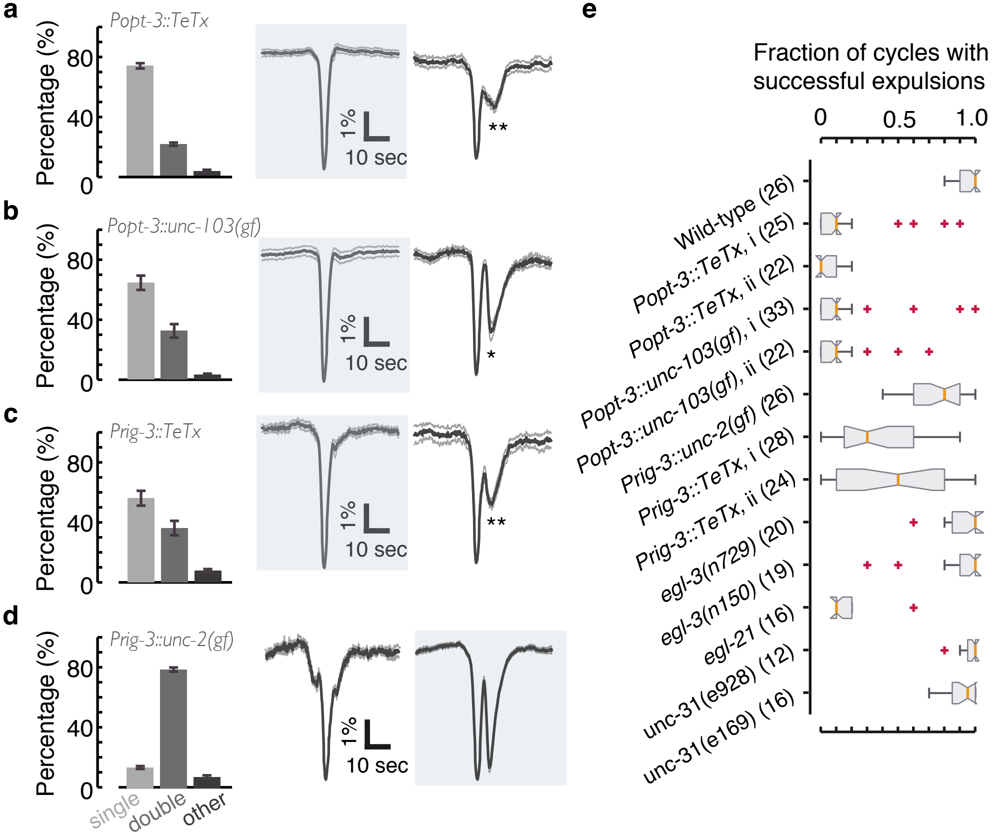

a
Popt-3::TeTx
Percentage (%)
80
40
0
1%
10 sec
**
b
Popt-3::unc-103(gf)
Percentage (%)
80
40
0
1%
10 sec
*
c
Prig-3::TeTx
Percentage (%)
80
40
0
1%
10 sec
**
d
Prig-3::unc-2(gf)
Percentage (%)
80
40
0
1%
10 sec
single
double
other
e
Fraction of cycles with successful expulsions
0
0.5
1.0
Wild-type (26)
Popt-3::TeTx, i (25)
Popt-3::TeTx, ii (22)
Popt-3::unc-103(gf), i (33)
Popt-3::unc-103(gf), ii (22)
Prig-3::unc-2(gf) (26)
Prig-3::TeTx, i (28)
Prig-3::TeTx, ii (24)
egl-3(n729) (20)
egl-3(n150) (19)
egl-21 (16)
unc-31(e928) (12)
unc-31(e169) (16)

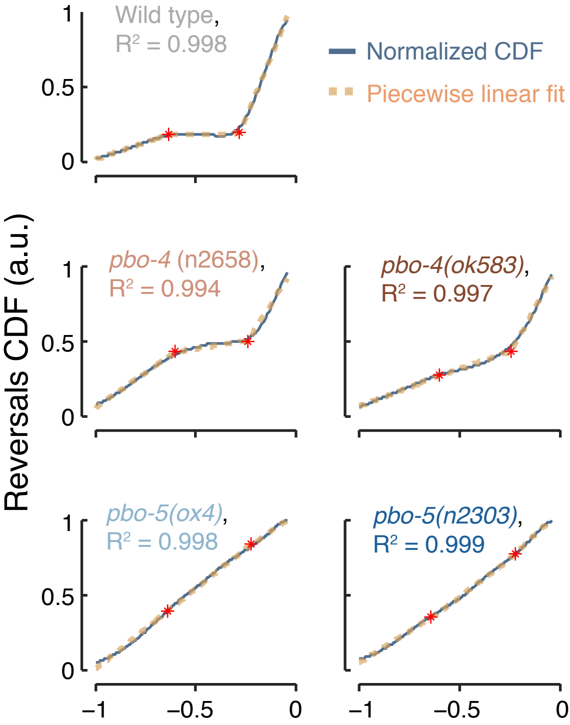

Wild type,
R^2 = 0.998
Normalized CDF
Piecewise linear fit
Reversals CDF (a.u.)
pbo-4 (n2658),
R^2 = 0.994
pbo-4(ok583),
R^2 = 0.997
pbo-5(ox4),
R^2 = 0.998
pbo-5(n2303),
R^2 = 0.999
0
0.5
1
−1
−0.5
0

Figure S1. Body contractions and detected cycles in DMP deficient mutants. Same as Fig. 1B-C for strains with known DMP defects and for *egl-3* and *egl-21* mutants. From left to right: N(wild-type) = 28, N(*egl-8*) = 9, N(pbo-4) = 11, N(pbo-5) = 13, N(*unc-16*) = 10, N(*egl-3*) = 10, N(*egl-21*) = 10, N(*unc-31(e928)*) = 14, N(*unc-31(e169)*) = 9, and N(*aex-5*) = 11 animals. Wild-type data was reproduced from Fig. 1B-C for convenience. When single or double contractions were detected at a frequency lower than 15% the resulting mean body-length could have been significantly affected by experimental noise. Here, these cases are denoted as "N/A".

Figure S2. Timing of DMP cycles in mutants with altered PKA activity. The periods between DMP cycles (left) and the durations of epochs between Bocs within a cycle (right) for mutants in which PKA activity was altered. Wild-type data was reproduced from Fig. 1. N(*kin-2*) = 15, N(*acy-1(gf)*) = 15, and N(*acy-1(lf)*) = 13. Error bars depict mean ± s.e.m and single asterisks denote significant differences from wild-type ($p<0.05$).

Figure S3. The DMP and the DAMP co-reset. Same as Fig. 3**b**, but for a stimulus of 20mW blue light ($\lambda = 475 \pm 15$ nm), initiated at t = -15 sec and terminated at t = 0. As opposed to the mechanical stimulus, blue light either induced a DMP cycle during the stimulus or delayed it for approximately one period (t = 60 sec). Forward locomotion propensity aligned by the stimulus (bottom) was reproduced from [71].

Figure S4. The DMP and the DAMP co-terminate. (**a**) The body-lengths, anterior and posterior angles, and forward and backward locomotion of wild-type L4 lethargus during 10 minute period leading to and immediately after the onset of L4 lethargus. During the

first 10 minutes after the onset of lethargus, Bocs and DAMP posture dynamics were detected, DAMP backward motion was weakly evoked, and DAMP forward motion was not evoked. During the next 10 minute period, body contraction, posture dynamics, and locomotion dynamics were mostly associated with transitions from quiescence to motion, indicating that the DMP and the DAMP co-terminated. In all panels N = 28 animals and thin lines depict ±s.e.m. (**b**) The body-lengths of wild-type L4 lethargus larvae were aligned at the onset of bouts of quiescence (grey) or motion (brown). The data was plotted as a function of time (left) and of progression through the bout, i.e., time normalized by the duration of the bout (right). Thin lines depict ±s.e.m.

Figure S5. The dynamics of locomotion and posture during the DAMP of *pbo-4* and *pbo-5* mutants. Data was plotted in the same manner as in Fig. 2**a**, **b**. Top row: the likelihood of observing forward locomotion, backward locomotion, and dwelling during a DMP cycle. Bottom row: the mean magnitude of anterior, posterior, and mid-body angles (see methods). Locomotion and angle data were aligned at the trough of the pBoc (t = 0) before averaging. Wild-type data were reproduced from Fig. 2 for convenience. Thin lines depict mean ± s.e.m and double asterisks denote significant differences from wild-type (p<0.01).

Figure S6. (**a**) Left: the fractions of detected single, double, and multiple body-length troughs during the L4 stage of *Popt-3::TeTx* transgenics. Middle/right: the mean body length of *Popt-3::TeTx* transgenics during single- and double-trough events, respectively. N = 11 animals and error bars or thin lines depict mean ± s.e.m. (**b-d**) The same as panel (**a**) for *Popt-3::unc-103(gf)* (N=11), *Prig-3::TeTx* (N=9), and *Prig-3::unc-2(gf)*

(N=10), respectively. Double asterisks denote a significant difference from wild-type ($p<0.01$). (**e**) The fractions of DMP cycles with successful expulsions in the above described transgenics and in mutants with broad defects in peptidergic signaling. Box plot depicts outliers (red crosses), 75% and 25% quantiles (grey boxes), medians (orange lines), and remaining non-outlier data (dark lines).

Figure S7. Proton signaling affects the shaping of DAMP dynamics. Piecewise linear fits to the scaled cumulative propensities of backward motion during the 25 seconds preceding the aBoc. Data is shown for of wild-type animals, *pbo-4* mutants, and *pbo-5* mutants. Comparisons of the resulting slopes are presented in Fig. 7**b**.

Supplementary movie M1. An example defecation cycle. Red arrows denote the timing of the pBoc and the aBoc. Duing the pBoc the posterior body-wall muscles contract symmetrically and relaxes asymmetrically as backward motion is initiated. Backward motion is terminated upon the initiation of the aBoc, after which forward locomotion is ensues. The movie is slowed down two fold with respect to real time.
